# Relationship between Serum Bortezomib Concentration and Emergence of Diarrhea in Patients with Multiple Myeloma and/or AL Amyloidosis

**DOI:** 10.3390/cancers13225674

**Published:** 2021-11-12

**Authors:** Yuki Fujimoto, Shikiko Ueno, Kazutaka Oda, Nao Gunda, Yumi Shimomura, Yuka Nishimura, Ayami Yamaguchi, Akari Kuwano, Yuki Ito, Yusuke Baba, Aina Nishigaki, Natsumi Michiwaki, Shota Uchino, Kayo Kurogi, Yawara Kawano, Masao Matsuoka, Hideyuki Saito, Yutaka Okuno, Hirofumi Jono

**Affiliations:** 1 Department of Clinical Pharmaceutical Sciences, Graduate School of Pharmaceutical Sciences, Kumamoto University, Kumamoto 860-8556, Japan; fujimoto.yuuki.nb@mail.hosp.go.jp (Y.F.); naog@almeida.oita.med.or.jp (N.G.); hiyo_no_mail4hatoress@yahoo.co.jp (Y.S.); nyuuu.lzlo@gmail.com (Y.N.); aasanc5s2@gmail.com (A.Y.); kinakoromochi_1212@yahoo.co.jp (A.K.); 161p1003@st.kumamoto-u.ac.jp (Y.I.); 161p1043@st.kumamoto-u.ac.jp (Y.B.); 162p1039@st.kumamoto-u.ac.jp (A.N.); 160p1052@st.kumamoto-u.ac.jp (N.M.); 175p1008@st.kumamoto-u.ac.jp (S.U.); 178p1015@st.kumamoto-u.ac.jp (K.K.); saitohide@kuh.kumamoto-u.ac.jp (H.S.); 2 Department of Pharmacy, National Hospital Organization Beppu Medical Center, Beppu 874-0011, Japan; 3 Department of Hematology, Rheumatology, and Infectious Disease, Kumamoto University Hospital, Kumamoto 860-8556, Japan; shikiko-u@kuh.kumamoto-u.ac.jp (S.U.); ykawanomn@kuh.kumamoto-u.ac.jp (Y.K.); mamatsu@kumamoto-u.ac.jp (M.M.); 4 Department of Pharmacy, Kumamoto University Hospital, Kumamoto 860-8556, Japan; kazutakaoda@kuh.kumamoto-u.ac.jp

**Keywords:** multiple myeloma, bortezomib, diarrhea, serum concentration, discontinuation

## Abstract

**Simple Summary:**

Multiple myeloma patients have benefited from bortezomib therapy, though it has often been discontinued owing to diarrhea. The objective of this study was to verify serum bortezomib concentration in the emergence of diarrhea. Twenty-four patients with bortezomib therapy were recruited; eight patients (33.3%) developed diarrhea at day 3 as median. The median measured trough bortezomib concentration at 24 h after first or second dose for patients with or without diarrhea was 0.87 or 0.48 ng/mL, respectively (*p =* 0.04, Wilcoxon signed rank test). Receiver operation characteristic (ROC) analysis produced the cut-off concentration of 0.857 ng/mL (area under the ROC curve of 0.797, sensitivity of 0.625, specificity of 0.875). In conclusion, this study indicated the possible involvement of serum bortezomib concentration in the emergence of diarrhea in bortezomib therapy in patients with multiple myeloma.

**Abstract:**

(1) Background: multiple myeloma patients have benefited from bortezomib therapy, though it has often been discontinued owing to diarrhea. The objective of this study was to verify serum bortezomib concentration in the emergence of diarrhea. (2) Methods: this prospective, observational case-control, and monocentric study was performed with an approval by the Ethics Committee of Kumamoto University Hospital in 2015 (No. 1121) from February 2015 to April 2017. (3) Results: twenty-four patients with bortezomib therapy were recruited; eight patients (33.3%) developed diarrhea at day 3 as median. Median measured trough bortezomib concentration at 24 h after first or second dose for patients with or without diarrhea was 0.87 or 0.48 ng/mL, respectively (*p =* 0.04, Wilcoxon signed rank test). Receiver operation characteristic (ROC) analysis produced the cut-off concentration of 0.857 ng/mL (area under the ROC curve of 0.797, sensitivity of 0.625, specificity of 0.875). The survival curves between patients with and without diarrhea were similar (*p =* 0.667); those between patients with higher and lower concentration than median value (0.61 ng/mL) were also similar (*p =* 0.940). (4) Conclusions: this study indicated the possible involvement of serum bortezomib concentration in the emergence of diarrhea in bortezomib therapy in patients with multiple myeloma.

## 1. Introduction

Multiple myeloma is the plasma cell related cancer, occupying 10% of all hematologic cancers and 1% of all cancers [1,2,3,4]. Historically, multiple myeloma had lacked an effective therapeutic strategy; the median overall survival had been less than 30 months [5]. The therapeutic objective of anticancer agents before the 1990s was to maintain stability or plateau disease. Thereafter, thalidomide was used as the effective agent, and then complete response could be targeted in a small number of populations [6]. Since bortezomib was then approved as the first effective proteasome inhibitor for multiple myeloma by the US Food and Drug Administration (FDA) in 2003, the median overall survival has been improved to more than 55 months [7]. A meta-analysis demonstrated that a bortezomib-based treatment regimen significantly benefited about overall survival, progression-free survival, and response rate [4,8,9,10]. Therefore, bortezomib is still a pivotal drug in the treatment of multiple myeloma.

However, bortezomib therapy has often been discontinued owing to some side effects, as represented by gastrointestinal toxicity, fatigue, peripheral neuropathy, and thrombocytopenia [11]. Gastrointestinal toxicity is one of the major concerns; the emergence rate was up to 84% [12]. Diarrhea can emerge in 44–51% of all grades, 7% of grade 3 and <1% of grade 4 [13,14]. Such severe diarrhea is introduced as dose-limiting toxicity requiring appropriate dose reduction in a bortezomib dosing manual without an appropriate pharmacokinetic exposure index [15,16,17]. Although the mechanism for the emergence of diarrhea in bortezomib therapy remains unknown, serum bortezomib concentration can be hypothesized to be attributed to the emergence of diarrhea based on pharmacokinetic/pharmacodynamics principles.

The purpose of this study is to verify the possible involvement of serum bortezomib concentration in the emergence of diarrhea in patients with multiple myeloma, and investigate a safer and efficient dosing strategy for bortezomib therapy.

## 2. Materials and Methods

### 2.1. Study Design, Ethics and Patients

The prospective, observational case-control, and monocentric study was performed in accordance with the Declaration of Helsinki and national and institutional standards after approval by the Ethics Committee of Kumamoto University Hospital in 2015 (No. 1121) from February 2015 to April 2017. The study was also conducted in accordance with the Basic and Clinical Pharmacology and Toxicology policy for experimental and clinical studies [18]. Written informed consent was obtained from every recruited patient by a document. All patients who required bortezomib therapy for the treatment of multiple myeloma were recruited. Diarrhea could emerge prior to reaching a pharmacokinetically steady state and trough concentration (72 h after dosing) in initial stage of dosing could be under lower limit of quantification. Therefore, an attending physician harvested blood samples from participants at 24 h after first dose (if missed, after second dose) of bortezomib, when the concentrations could be measured in small deviation and reached over end of the distribution phase then into the elimination phase [7]. The blood samples were centrifuged 4 min in 3000× *g* at room temperature to obtain sera, and then stored using a 1.5 mL plastic tube until measurement at −30 °C in a deep freezer.

### 2.2. Outcomes Measures

Emergence of diarrhea was employed as a primary outcome defined by Common Terminology Criteria for Adverse Events (CTCAE) version 5.0 at any grading [19]. Time after bortezomib dose to emergence of diarrhea was analyzed by Kaplan–Meier curve. Median serum concentration of patients with diarrhea was compared with that without diarrhea. We then tried to calculate the cut-off value of serum concentration for the emergence of diarrhea using receiver operating characteristic (ROC) analysis [20]. Physiological and basic status of patients were compared between the groups to identify potential factors for the emergence of diarrhea, then a multivariate logistic regression analysis was considered regarding the identified potential factors with statistical and clinical significance. Survival rate was employed as a secondary outcome in being stratified by the emergence of diarrhea or identified factors for diarrhea.

### 2.3. Development of Serum Bortezomib Concentration Measurement Method

Bortezomib for the standard reagent was purchased from Selleck Chemicals (Houston, TX, USA). Bortezomib-d8 as an internal standard was purchased from Toronto Research Chemicals (Toronto, ON, Canada). The measurement was performed using a liquid chromatography-tandem mass spectrometer (LC-MS/MS). The part of liquid chromatography consisted of the Prominence UFLC (SHIMADZU, Kyoto, Japan), where the separation column was equipped with the Atlantis^®^C18 (3 µm, 2.1 × 50 mm, Waters, Milford, CT, USA). The part of tandem mass spectrometer was the API3200 of Sciex (Framingham, MA, USA).

Serum samples were resolved in room temperature for purification prior to measurement, then sera of 100 µL of were prepared. Acetonitrile of 500 µL with 0.1% formic acid including bortezomib-d8 were added to the sera, then the mixtures were centrifuged 10 min at 13,000× *g* on 4 °C by following 10 s-vortex. The supernatants of 400 µL were evaporated and concentrated in a decompression chamber for 3 h on 35 °C, then the residuals were resolved in acetonitrile of 20 µL with 0.1% formic acid. The acetonitrile solutions were injected by an autosampler.

Separation settings were as follows: mobile phase A was milli Q water with 0.1% formic acid, mobile phase B was acetonitrile with 0.1% formic acid, the temperature was set to 50 °C, the flow rate was 0.3 mL/min. The separation was performed in a gradient mode with total of 10 min for one cycle; the first rate of mobile phase A was 5% and maintained until 0.5 min, then increased to 95% over next one minute (until 1.5 min), maintained until 7 min, and decreased to 5% over next 0.5 min (until 7.5 min) then maintained until 10 min for stabilizing in the initial mobile phase A ratio. Polarity, scan type and ion source were “Positive”, “MRM” and “Turbo spray”, where the parameters were CUR: 10.00, IS: 5000.00, TEM: 600.00, GS1: 70.00, GS2: 30.00, ihe: ON. The m/z of bortezomib was 367.1–226.0, with DP: 65.00, EP: 2.00 CE: 24.00 CXP: 0.00.

The calibration curve was prepared using the concentrations of 0.16, 0.32, 0.64, 1.25, 2.5, 5.0, and 10.0 ng/mL. Intra- or inter-day (serial 5 days) variability was evaluated at three quality control (QC) concentrations of 0.3125, 2.5, and 10 ng/mL. Precision and accuracy were evaluated by equations as follows:

Precision (%) = (standard deviation for measured value/mean measured value) × 100

Accuracy (%) = (Mean measured value/prepared concentration) × 100

Recovery rate, extraction rate, and matrix effect were then examined.

### 2.4. Statistical Analysis

Every statistical calculation was performed using R ver. 3.6.2 (https://www.r-project.org/, accessed on 28 December 2019), where a *survfit* function in a *survival* package was employed for the Kaplan–Meier curve analysis; a *prediction* function *ROCR* package was employed for the ROC analysis. Every statistical evaluation between groups employed the paired t-test or the Wilcoxon rank test for continuous variables and Fisher’s exact test for categorical variables. *p* value < 0.05 was settled for the statistical significance.

## 3. Results

### 3.1. Development of Serum Bortezomib Concentration Measurement Method

**Figure 1:**
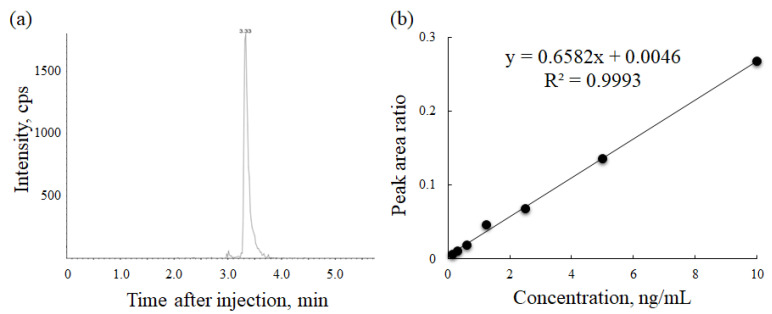
Development of serum bortezomib concentration measurement method. (**a**) The chromatogram in the concentration of 10.0 ng/mL; (**b**) the calibration curve in the range from 0.16–10.0 ng/mL.

The chromatogram for the serum concentration of 10 ng/mL bortezomib is provided in Figure 1a. The retention time was 3.3 min for both bortezomib and the internal standard. The calibration curve is shown in Figure 1b. Linearity was confirmed within the range of the concentrations (0.16–10 ng/mL). The intra-day and inter-days variabilities indicated that the all values of accuracy were within +/− 15% (Supplementary Table S1), and the maximum value of precision was 7.2%.

### 3.2. Relationship between Serum Bortezomib Concentration and Emergence of Diarrhea

**Figure 2:**
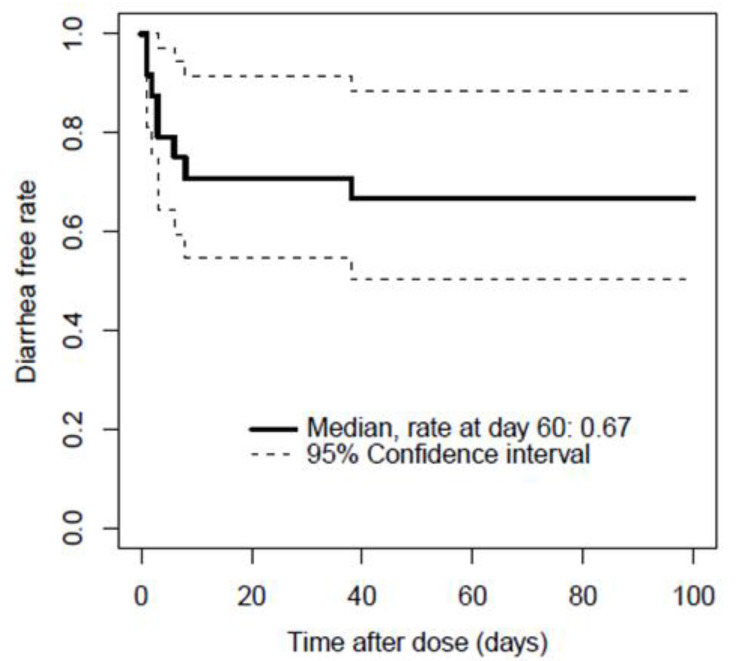
Time between bortezomib dosing and emergence of diarrhea. Median days for the emergence was three; diarrhea free rate at day 60 was 0.67.

This study included 24 patients with bortezomib therapy where eight patients developed diarrhea (33.3%, grade 1, four patients; grade 2, four patients). Patients who had diarrhea discontinued bortezomib therapy or continued same dose prior to discontinuation. No patients reduced the dose for continuation and measured again. Demographics of patients with and without diarrhea are shown in Table 1. None of three patients with gastrointestinal amyloidosis developed diarrhea. Two of seven patients with peripheral neuropathy developed diarrhea. Age, blood urea nitrogen, measured serum bortezomib concentration, and ratio of concentration/dose presented statistical significances between the groups. Moreover, the serum bortezomib concentration was correlated with the concentration/dose and age. Since low blood urea nitrogen was considered to be clinically meaningless for the emergence of diarrhea, multivariate logistic regression analysis was consequently not performed. The results shown in Figure 2 indicated that the Kaplan–Meier curve described the time after bortezomib dose to the emergence of diarrhea at day 3 as median; diarrhea-free rate at day 60 was 0.67. Time between bortezomib dosing and emergence of diarrhea stratified by grading were described in Supplementary Figure S1.

**Figure 3:**
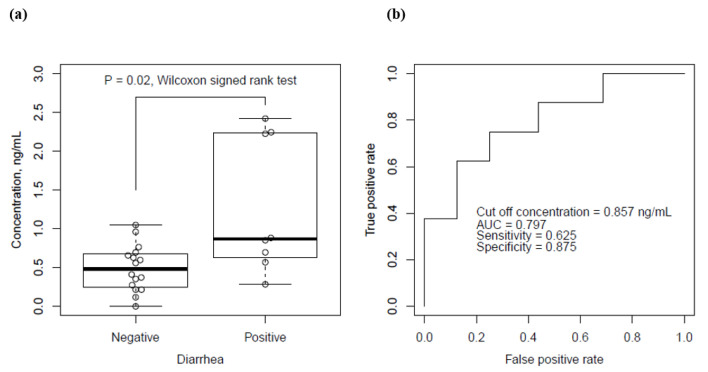
Association of serum bortezomib concentration with the emergence of diarrhea. (**a**) Boxplots of serum bortezomib concentration in patients without or with diarrhea; (**b**) receiver operation characteristic (ROC) analysis of serum bortezomib concentration for the emergence of diarrhea.

Serum bortezomib concentration was measured in 24 recruited patients. The median value in patients with or without diarrhea was significantly different at 0.87 (interquartile: 0.66–2.23, *p* < 0.020) ng/mL or 0.48 (interquartile: 0.26–0.67) ng/mL, respectively (Table 1, Figure 3a). No apparent relationship between the dose and the measured concentration of bortezomib was observed. ROC curve analysis produced the cut-off concentration of 0.857 ng/mL for the emergence of diarrhea with sensitivity of 0.625, specificity of 0.875, and area under the ROC curve of 0.797 (Figure 3b). The median (interquartile) values for patients with/without neuropathy, thrombocytopenia, leukopenia, or anemia were 0.659 (0.483–0.778)/0.564 (0.274–0.879) ng/mL, 0.600 (0.348–0.700)/0.696 (0.290–0.922) ng/mL, 0.405 (0.316–0.661)/0.696 (0.553–0.879) ng/mL, or 0.559 (0.300–0.747)/0.758 (0.614–0.874) ng/mL (*p* values were 0.764, 0.839, 0.976, or 0.597, respectively).

**Figure 4:**
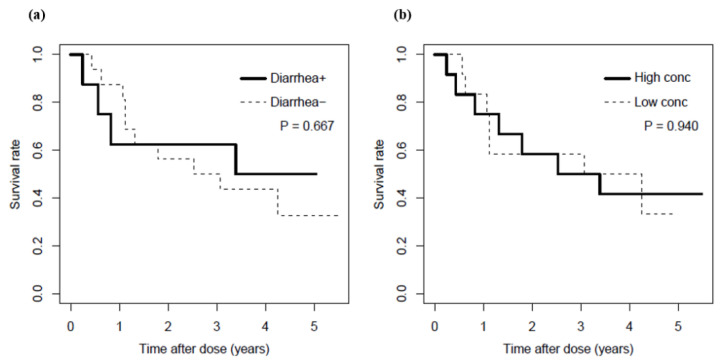
Survival curves in patients with bortezomib therapy. (**a**) Survival curves in the patients with (bold line) or without (dashed line) diarrhea; (**b**) survival curves in patients with higher concentration (>median, bold line) or lower concentration (≤median, dashed line). The median concentration was 0.61 ng/mL.

Survival rate was evaluated as a secondary outcome (Figure 4). Patients with diarrhea provided a similar survival rate–time curve to those without diarrhea (*p =* 0.667, Figure 4a). The recruited patients were further allocated in accordance with serum bortezomib concentration higher/lower than median value of 0.61 ng/mL. The patients with the higher concentration provided similar survival rate–time curve with those with the lower concentration (*p =* 0.940, Figure 4b).

## 4. Discussion

To address the issue of diarrhea as a cause of discontinuing bortezomib therapy in patients with multiple myeloma, we showed that serum bortezomib concentration was possibly involved in the emergence of diarrhea. To the best of our knowledge, this study was the first report to advocate for a safer bortezomib dosing technique and that the serum bortezomib concentration of 0.857 ng/mL may be the target value for reducing the risk of diarrhea by ROC analysis. Although the mechanism of diarrhea caused by proteasome inhibitors has yet to be revealed, this study suggests the possible involvement of serum bortezomib concentration in the emergence of diarrhea. Previous studies demonstrated that most of the trough concentration was <1 ng/mL by a dose of 1.0 or 1.3 mg/m^2^ [21,22]. The cut-off value of 0.857 ng/mL was in the middle of normally measured concentrations; it is understandable that up to 84% of patients with bortezomib therapy can induce gastrointestinal side effects including diarrhea by bortezomib [12]. Because one dose or two doses is/are sufficient for the emergence of diarrhea, serum bortezomib concentration measurement may be suboptimal and late to avoid diarrhea. Therefore, the first dose reduction should be considered for patients with probable high bortezomib concentration; however, potential risk factors for high bortezomib concentration remain unclear in this study. The sensitivity, specificity, and AUC by the ROC analysis were low. Figure 3a showed only three out of eight patients with diarrhea demonstrated high concentration of bortezomib. The remaining five patients showed almost the same concentration as diarrhea-negative patients. Severity of diarrhea was equally recoded as grade 1 or 2 for a respective four patients, where no association between the severity and serum concentration was observed. Therefore, the cut-off value must be further investigated.

Bortezomib is metabolized in liver microsomes [23]; liver dysfunction could be a potential risk for high bortezomib concentration [24]. As the previous study demonstrated that total bilirubin level as a surrogate marker for cirrhosis was associated with high bortezomib concentration [24], this study also indicated a potential of total bilirubin level for the risk factor, but the statistical significance was not indicated (Table 1). Other possible surrogate markers for liver dysfunction, such as low serum albumin, high/low transaminases, and low cholinesterase were also not significantly correlated in this study. More specifically, most of bortezomib is metabolized by cytochrome *p* 450 (CYP) 2C19 or 3A4 [22]. Polymorphism in CYP2C19, where common mutant alleles are CYP2C19*2 and CYP2C19*3, has been known to reduce the metabolic capability. The frequency in Asian populations is 30–50% and 5–10%, respectively [25,26]. Coadministration of omeprazole (a proton pump inhibitor), a popular inhibitor of CYP2C19, unexpectedly maintained bortezomib concentration [27]. In this study, the seven patients with higher concentration than the cut-off took a proton pump inhibitor (lansoprazole 3, rabeprazole 3, esomeprazole 1), while the remaining 17 patients took that (lansoprazole 6, rabeprazole 1, esomeprazole 6, vonoprazan 1), where any statistical significance were unidentified. Interestingly, poor metabolizers of CYP2C19 reportedly retained treatment efficacy [28]; standard dosing regimen and the corresponding serum bortezomib concentration may be sufficient for the maximum therapeutic efficacy. This study also indicated the comparable survival curve in patients with high and low concentration (Figure 4b). Importantly, coadministration of rifampicin, a typical inducer of CYP3A4, reportedly decreased bortezomib concentration, but the therapeutic efficacy was maintained [29], whereas a typical inhibitor of CYP3A4 possibly exacerbates adverse reactions induced by bortezomib [30]. Collectively, appropriate dose reduction may contribute to a safer dosing regimen accompanied by maintained therapeutic efficacy.

In clinical practice, the fact that diarrhea was caused by the administration of bortezomib may indicate that its serum concentration is high, therefore it is reasonable to reduce the dose in these patients after the first administration. By reducing the dose, it can be expected that bortezomib treatment will be completed without causing diarrhea (adverse events) thereafter. The phase III VISTA study by Mateos et al. suggests that the cumulative dose of bortezomib is associated with overall survival [31]. Therefore, in order to obtain the expected therapeutic effect of bortezomib, continuous long-term administration is required without discontinuation due to adverse events. Our analysis supports that when diarrhea develops, the dose of bortezomib should be reduced appropriately without hesitation, and as a result, achievement of long-term continuation should lead to improved prognosis.

This study involves some limitations. First, this study was not designed to identify potential factors associated with bortezomib concentration. The sample size was relatively small for the purpose. Second, we employed trough concentration for the pharmacokinetic index of diarrhea, other exposure indexes, such as peak concentration or area under the concentration–time curve, might be pivotal. Third, overt heterogeneity was not observed between the groups in Table 1, possible heterogeneities regarding other characters might be hidden owing to this case-control study design.

## 5. Conclusions

This study indicated the possible involvement of serum bortezomib concentration in the emergence of diarrhea in patients with multiple myeloma. The possible exposure dependency may suggest first dose reduction supported by factors associating with bortezomib concentration, where the treatment efficacy may be preserved.

## Figures and Tables

**Table 1:** Demographics of recruited patients.

Character		Patients without Diarrhea		Patients with Diarrhea		*p* Value
Baseline characteristics						
Male/female (male %)		11/5 (68.8%)		4/4 (50%)		0.412
Age, years		77	(49–83)	62	(54–71)	0.008 *
Height, cm		160.9	(145.1–171.2)	162.1	(140.0–167.4)	0.811
Weight, kg		57.4	(43–78.4)	60.4	(38.6–71.2)	0.893
Serum albumin, g/dL		3.3	(1.3–5.9)	3.8	(2.5–4.6)	0.579
Blood urea nitrogen, mg/dL		19.3	(11.4–70.4)	12.8	(6.8–23.1)	0.033
Serum creatinine, mg/dL		0.97	(0.42–8.01)	0.74	(0.47–1.33)	0.229
Total bilirubin, mg/dL		0.55	(0.30–1.00)	0.65	(0.40–2.30)	0.195
Alanine transaminase, IU/L		19	(11–68)	22.5	(14–66)	0.475
Aspartate transaminase, IU/L		22	(9–90)	17	(10–54)	0.696
γ-glutamyl transpeptidase, IU/L		24	(7–125)	29	(11–343)	0.427
Cholinesterase, IU/L		237	(102–451)	244	(113–321)	0.634
White blood cell, counts × 10^3^/μL		5	(2.9–8.7)	3.5	(1.6–10.1)	0.387
Red blood cell, counts × 10^6^/μL		3.7	(2.7–5.0)	3.5	(2.8–4.9)	0.773
Hemoglobin, g/dL		11.8	(8.6–15.4)	9.9	(0.8–15.7)	0.377
Hematocrit, %		36.1	(27.4–43.7)	33.7	(22.9–47.7)	0.993
Platelet, counts × 10^3^/μL		190	(56–315)	159	(53–246)	0.424
Prothrombin time, %		94	(11–134)	90	(61–137)	0.605
Prothrombin time-international normalized ratio		1.02	(0.89–1.76)	1.05	(0.89–1.26)	0.610
Comorbidities						
	Hypertension (%)	7 (43.8%)		3 (37.5%)		1.000
	Chronic kidney disease (%)	6 (37.5%)		0 (0.0%)		0.066
	Diabetes mellitus (%)	2 (12.5%)		2 (25.0%)		0.578
	Solid carcinoma (%)	3 (18.8%)		1 (12.5%)		1.000
	Heart failure (%)	7 (43.8%)		2 (25.0%)		0.657
	Liver disiease (%)	7 (43.8%)		2 (25.0%)		0.657
	Alcohol consumption	5 (31.3%)		3 (37.5%)		1.000
Multiple myeloma type						0.674
	IgGκ (%)	6 (37.5%)		4 (25.0%)		
	IgGλ (%)	0 (0.0%)		1 (6.3%)		
	IgAκ (%)	1 (6.3%)		1 (6.3%)		
	IgAλ (%)	2 (12.5%)		0 (0%)		
	AL amyloidosis (%)	4 (25.0%)		1 (6.3%)		
	Ohters (%)	3 (18.8%)		1 (6.3%)		
Recurrence (%)		4 (25.0%)		4 (50.0%)		0.363
Gastrointestinal amyloidosis		3 (18.8%)		0 (0%)		0.526
Disease stage						
	I	7 (43.8%)		7 (87.5%)		0.079
	II	5 (31.3%)		1 (12.5%)		0.621
	III	4 (25.0%)		0 (0.0%)		0.262
Exposure						
Measured concentration, ng/mL		0.48	(0.26–0.67)	0.87	(0.66–2.23)	0.040 *
Measurement after second dose		4 (25.0%)		3 (37.5%)		0.866
Dose, mg/m^2^						0.657
	1.3 mg/m^2^ (%)	9 (56.2%)		6 (75.0%)		
	1.0 mg/m^2^ (%)	7 (43.8%)		2 (25.0%)		
Body surface area, m^2^		1.6	(1.3–1.9)	1.6	(1.3–1.8)	1.000
Dose, mg		1.7	(1.5–2.4)	1.9	(1.6–2.2)	0.433
Concentration/dose, (ng/mL)/(mg/m^2^)		0.5	(0.0–1.0)	0.8	(0.3–1.9)	0.020
Injection route						
	Subcutaneous injection (%)	15 (93.8%)		8 (100%)		1.000
	Intravenous injection (%)	1 (6.3%)		0 (0.0%)		1.000
Concomitant CYP 2C19 inhibitors						
	Lansoprazole (%)	7 (43.8%)		2 (25.0%)		0.657
	Esomeprazole (%)	5 (31.3%)		2 (25.0%)		1.000
	Rabeprazole (%)	1 (6.3%)		3 (37.5%)		0.091
	Vonoprazan (%)	1 (6.3%)		0 (0.0%)		1.000
	Fluconazole (%)	1 (6.3%)		0 (0.0%)		1.000
Concomitant CYP 3A4 inhibitors						
	Dexamethasone (%)	15 (93.8%)		7 (87.5%)		1.000
Concomitant CYP 3A4 inducers						
	Amlodipine (%)	1 (6.3%)		3 (37.5%)		0.091
	Fluconazole (%)	1 (6.3%)		0 (0.0%)		1.000
	Others (%)	3 (18.8%)		1 (12.5%)		1.000
Regimens						
	Bor + CPA + DEX (%)	12 (75.0%)		5 (62.5%)		0.647
	Bor + DEX (%)	2 (12.5%)		1 (12.5%)		0.333
	Others (%)	2 (12.5%)		2 (25.0%)		0.578
Outcomes						
Peripheral neuropathy (%)		4 (25.0%)		3 (37.5%)		0.647
Fatigue (%)		1 (6.3%)		1 (12.5%)		1.000
Nausea or vomiting (%)		1 (6.3%)		0 (0.0%)		1.000
Constipation (%)		9 (56.3%)		1 (12.5%)		0.079
Thrombocytopenia (<75,000 counts/μL%)		8 (50%)		5 (62.5%)		0.679
Fever (>38 °C) (%)		1 (6.3%)		1 (12.5%)		1.000
Anemia (Hb <10.0 g/dL) (%)		12 (75%)		6 (75%)		1.000
Leukopenia (<3,000 counts/μL)%		8 (50%)		3 (37.5%)		0.680
Response rate (%)		12 (75.0%)		4 (50.0%)		0.363

Continuous variables were presented by median (minimum–maximum). Response rate included patients with partial response or molecular response or complete response. Complications with CTCAE ver. 5.0 at any grading were summarized. *Bor* bortezomib, *CPA* cyclophosphamide, *DEX* dexamethasone. * Statistical significance.

## Data Availability

The data presented in this study are available on request from the corresponding author.

## Supplementary Materials

The following is available online at https://www.mdpi.com/article/10.3390/cancers13225674/s1: Table S1. Intra-day and inter-days variabilities of measurement method; Figure S1. Time between bortezomib dosing and emergence of diarrhea stratified by grading. Left, diarrhea grade 1. Right, diarrhea grade 2.
