# Supplementary material for: Relationship between Serum Bortezomib Concentration and Emergence of Diarrhea in Patients with Multiple Myeloma and/or AL Amyloidosis
Source: Cancers (Basel). 2021 Nov 12;13(22):5674. doi: 10.3390/cancers13225674 (PMC8616141; doi:10.3390/cancers13225674)
Supplement: Supplementary file 1 [file cancers-13-05674-s001.zip › cancers-1423878-supplementary.pdf]

Supplementary Table S1. Intra-day and inter-days variabilities of measurement method.

	Intra-day variability (n=6)			Inter-days variability (n=5)		
Theoretical value ($ng\ mL^{-1}$)	Measured value ($ng\ mL^{-1}$)	Precision (%)	Accuracy (%)	Measured value ($ng\ mL^{-1}$)	Precision (%)	Accuracy (%)
0.3125	0.32 (0.02)	5.2	103	0.34 (0.01)	3.7	108
2.5	2.61 (0.11)	4.1	104	2.58 (0.11)	4.4	103
10	10.14 (0.73)	7.2	101	9.15 (0.42)	4.6	91

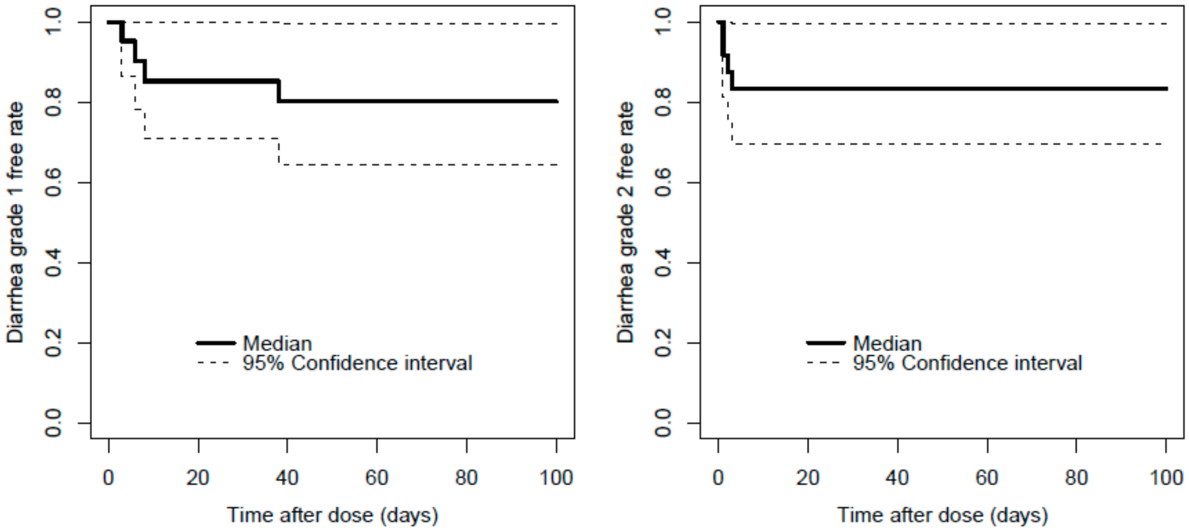

Supplementary Figure S1. Time between bortezomib dosing and emergence of diarrhea stratified by grading. Left, diarrhea grade 1. Right, diarrhea grade 2.
